# Biological Control of Tomato Gray Mold Caused by *Botrytis Cinerea* with the Entomopathogenic Fungus *Metarhizium Anisopliae*

**DOI:** 10.3390/pathogens9030213

**Published:** 2020-03-13

**Authors:** Most.Sinthia Sarven, Qiuyan Hao, Junbo Deng, Fang Yang, Gaofeng Wang, Yannong Xiao, Xueqiong Xiao

**Affiliations:** 1 Provincial Key Laboratory of Plant Pathology of Hubei Province, College of Plant Science and Technology, Huazhong Agricultural University, Wuhan 430070, Hubei, China; sinthiazoarder@gmail.com (M.S.); haoqiuyan@webmail.hzau.edu.cn (Q.H.); jksgo@mail.hzau.edu.cn (G.W.); xiaoyannong@mail.hzau.edu.cn (Y.X.); 2 Jingmen (China Valley) Academy of Agricultural Science, Jingmen 448000, Hubei, China; jm403372610@163.com (J.D.); YF760608@163.com (F.Y.)

**Keywords:** *Metarhizium anisopliae*, *Botrytis cinerea*, gray mold, biocontrol, conidia, postharvest

## Abstract

Gray mold disease caused by *Botrytis cinerea* is a devastating disease that leads to serious financial loss. In this study, the entomopathogenic fungus *Metarhizium anisopliae* that acts against the gray mold pathogen *B. cinerea* was evaluated. *M. anisopliae* produced a significant inhibition zone in front of the *B. cinerea* colony in the dual culture test. In addition, volatile organic compounds generated by *M. anisopliae* were shown to have an inhibitory effect on *B. cinerea* mycelia growth and reduced 41% of gray mold severity of postharvest tomatoes. The 10% concentration of the culture filtrate of *M. anisopliae* inhibited 88.62% of colony radial growth as well as 63.85% of sclerotia germination and all conidia germination of *B. cinerea*. Furthermore, the culture filtrate of *M. anisopliae* retained its inhibitory effect against the radial growth of *B. cinerea* even after heating for 15 min at 100 °C. Feasible mechanisms of *M. anisopliae* involved in the control of *B. cinerea* were explored, and it was demonstrated that the plasma membrane of *B. cinerea* conidia was damaged by the product of metabolism of *M. anisopliae*. In addition, after treating with culture filtrate of *M. anisopliae*, the *B. cinerea* phenotype was shown to be abnormal, and cell organelles of *B. cinerea* mycelia were damaged significantly. A significant control efficacy of *M. anisopliae* against tomato gray mold was detected on both the detached leaf assay (84.24%) as well as the whole plant (72.38%). In addition, a 78% reduction in tomato fruit mold was detected at a 10% treated concentration of *M. anisopliae*. These findings suggest that *M. anisopliae* possesses potential as a biocontrol agent against tomato gray mold in the greenhouse and during the postharvest stage.

## 1. Introduction

The phytopathogenic fungus *Botrytis cinerea* Pers. has a broad range of hosts including more than 200 crop species, causing gray mold [1]. It causes serious pre- as well as postharvest diseases in many economically important crops, such as tomato, strawberry, pear, cherry, eggplant, grape, and peppers. [2,3]. Among them, tomato gray mold is a devastating disease that causes serious financial losses worldwide [4,5]. The pathogen can infect tomato fruits and each part of the tomato plant either by injury after pruning and harvesting or direct penetration [6]. It also produces characteristic symptoms under favorable conditions, especially at low temperatures (10 to 25 °C) coupled with humid conditions [7]. Generally, the symptoms of the disease become visible later than host infection because the pathogen remains dormant for a long time inside the host. This pathogen also can survive in crop debris after the growing season as a mycelium along with or without conidia or sclerotia [8]. Furthermore, the resistant germplasm and resistance variety of tomato gray mold disease have not been screened worldwide. Therefore, it is tough to control gray mold disease of tomatoes. 

Over previous decades, the control of *B. cinerea* has depended mainly on chemical fungicides [9]. However, evidence reveals that *B. cinerea* has developed certain resistance to many fungicides, including anilinopyrimidines dicarboximides, benzamide, fenhexamid, diethofencarb, procymidone, pyrimethanil, and hydroxyanilide [10,11,12]. Furthermore, a *B. cinerea* strain has multiple types of fungicide resistance that can be found in cropland [13]. To date, abuse and excessive use of fungicides have caused environmental pollution and disruption of ecological ecosystems, as well as pose a danger to human health due to the high residual fungicides effect in tomato fruit [14,15]. Hence, there is an urgent demand for substitute measures that could safely and efficiently control tomato gray mold. Biological control is undoubtably a good option. Generally, microbial bioagents, for example, fungi, bacteria, and yeast, can effectively manage plant diseases through antibiosis [16,17,18], competition for places and nutrients, volatile compounds [19,20], parasitism [21], and initiation of plant defense [22].

*M. anisopliae* is a widely distributed entomopathogenic fungus with immense efficacy against various insect pests such as locusts, thrips, whiteflies, and ticks [23,24,25,26]. Recently, *Meterhizium* spp. also showed multifarious roles in the ecosystem as endophytes, plant growth promoters, and antagonists of plant pathogens [27]. *M. anisopliae* revealed differential expression of genes encoding hydrolytic enzymes, antimicrobial peptides, toxins, cell wall proteins, as well as other molecules [28]. Secondary metabolites generated by this fungus exhibit various insecticidal, anticancer, antioxidant, and antimicrobial properties. They have also been suggested as potential substitutes for the improvement of new bioactive agents [29,30]. If a fungal strain retains dual actions against insect pests and also plant pathogens, this would be a potent advantage for crop protection. Hence, the entomopathogenic fungus in the current study, *M. anisopliae*, was inspected for their antifungal activities against *B. cinerea*.

Few previous studies have presented the inhibitory effect of *M. anisopliae* on *B. cinerea* [31,32]. However, the in vivo biocontrol efficiency and possible mechanism of the inhibition process is still unknown. The present study assessed the antagonistic effects of *M. anisopliae* on *B. cinerea* using culture filtrate, crude extract, and volatile organic compounds (VOCs), as well as exploring the mechanisms of inhibition. In addition, the efficacy of *M. anisopliae* was evaluated for disease severity reduction by assessing on detached leaves, whole tomato plants, as well as ripe fruits after harvesting. Overall, this study showed that *M. anisopliae* could be a potential biocontrol agent against tomato gray mold disease introduced by *B. cinerea* during the plant growth stage and fruit storage period. In this context, the present study aims to determine the antifungal activity of *M. anisopliae* against *B. cinerea* under in vitro and in vivo conditions and to explore possible mechanisms of biocontrol. 

## 2. Results

### 2.1. The Antagonism of M. anisopliae against to B. cinerea

**Figure 1:**
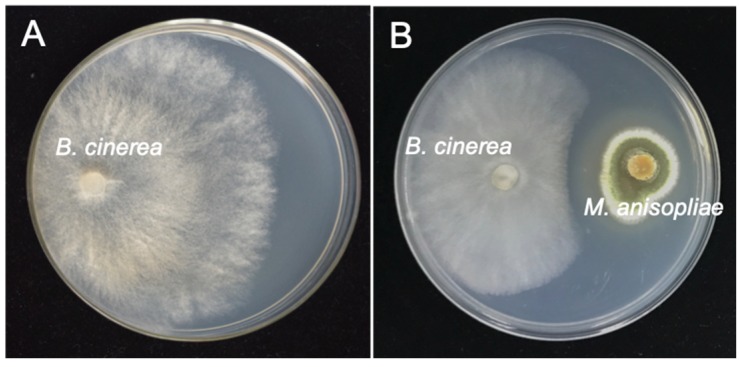
Inhibitory effect of *M. anisopliae* against *B. cinerea* in dual culture on potato dextrose agar. (**A**) Pure culture of *B. cinerea*, 20 °C, 4 dpi; (**B**) dual culture of *M. anisopliae* and *B. cinerea* showing inhibition zone between two colonies, 20 °C, 4 dpi.

Antagonistic assessment results showed that *M. anisopliae* has potential activity against *B. cinerea*. *M. anisopliae* produced a clear inhibition zone ahead of the *B. cinerea* colony (Figure 1). The average width of the inhibition zone was about 6.3 mm between two colonies, and *M. anisopliae* inhibited about 43.9% of *B. cinerea* radial growth as compared with the control. In addition, *B. cinerea* showed excessive twisted hyphal branching when inhibited by *M. anisopliae*. This indicates the existence of fungistatic secondary metabolites produced by *M. anisopliae*.

### 2.2. The M. anisopliae Culture Filtrate Influenced the Growth and Morphology of B. cinerea

**Figure 2:**
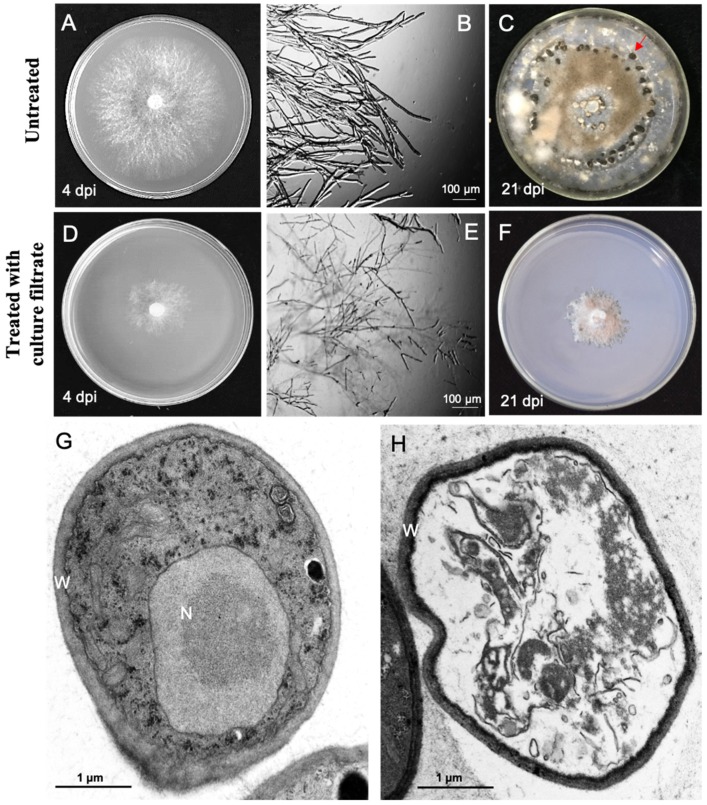
Inhibition activity of *M. anisopliae* culture filtrate on *B. cinerea* growth. (**A**–**C**) The normal phenotype of *B. cinerea* on potato dextrose agar (PDA); (**D**–**F**) abnormal growth of *B. cinerea* on PDA amended with 5% (*v*/*v*) culture filtrate of *M. anisopliae*. (**A**,**C**) colony of *B. cinerea* at 20 °C, 4 dpi; (**B**,**E**) hyphal tips of *B. cinerea;* (**G**,**H**) transmission electron microscopy (TEM) photos of the cells of *B. cinerea* mycelia. N represents the nucleus; W represents the cell wall. (**D**,**H**) colony of *B. cinerea* at 20 °C, 21 dpi; the arrow points to the sclerotium.

*B*. *cinerea* was cultured on potato dextrose agar (PDA) with added culture filtrate of *M. anisopliae*, and PDA plus potato dextrose broth (PDB) was used as the control. The colonies grown on PDA that amended 5% (*v*/*v*) culture filtrate were significantly smaller as compared with colonies grown on PDA (Figure 2A,D). Microscopic observation showed the mycelia of *B*. *cinerea* were vigorous, whereas culture filtrate treatment led the mycelia of *B*. *cinerea* with more short branches (Figure 2B,E). Transmission electron microscopy (TEM) observation exhibited that *B. cinerea* possess the normal structure of cell components in control treatments and intact and well-preserved cell walls, obvious nucleus, and mitochondria were frequently visible in the cells (Figure 2G). However, after treated with culture filtrate of *M. anisopliae* (5%, *v*/*v*), cell organelles were seriously damaged (Figure 2H). In addition, no sclerotium was observed at 21 dpi after treating *B*. *cinerea* with 5% (*v*/*v*) culture filtrate of *M. anisopliae* (Figure 2C,F). This suggests that the metabolites of *M. anisopliae* are able to suppress the mycelium growth and sclerotium formation of *B*. *cinerea*. 

**Figure 3:**
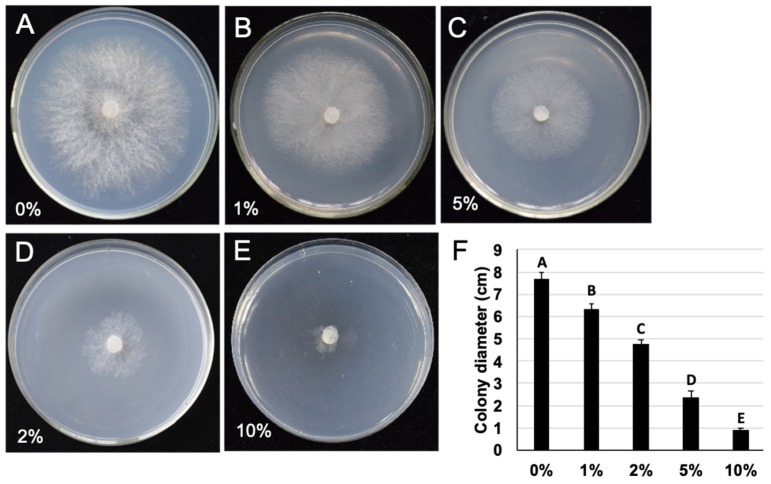
(**A**–**E**) colony of *B. cinerea* on PDA added to 0%, 1%, 2%, 5%, and 10% culture filtrates of *M. anisopliae*, 20 °C, 4 dpi; (**F**) colony diameter of *B. cinerea* on PDA added with different concentrations of culture filtrate, 20 °C, 4 dpi.

The inhibition rates of *M. anisopliae* culture filtrate to *B. cinerea* significantly (*P* < 0.01) increased with an increase in concentration of *M. anisopliae* culture filtrates (Figure 3). Among the tested concentrations of culture filtrate, culture filtrate at a concentration of 10% showed 86.7% growth inhibition to *B. cinerea*, and an inhibition rate of 68.9% was recorded when treated with culture filtrate at a concentration of 5%. Eventually, culture filtrate at a concentration of 1% showed an inhibitory rate of 19.42% to *B. cinerea* radial growth.

### 2.3. The Culture Filtrate of M. anisopliae Affected the Sclerotia Germination of B. cinerea 

Sclerotia that can survive in unfavorable environmental conditions are a resting form of *B. cinerea*; thus, we tested the influence of *M. anisopliae* on *B. cinerea* sclerotia germination. The sclerotia germination rate was 35.04% in the group treated with 10% culture filtrate, while the germination rate was 96.89% in the untreated control group (Table 1). The results showed that the inhibition rate of 10% culture filtrate to sclerotia germination reached 63.85%. This demonstrates that the culture filtrate of *M. anisopliae* can suppress the sclerotia germination of *B. cinerea*.

### 2.4. The Culture Filtrate of M. anisopliae Inhibited Conidia Germination of B. cinerea

**Figure 4:**
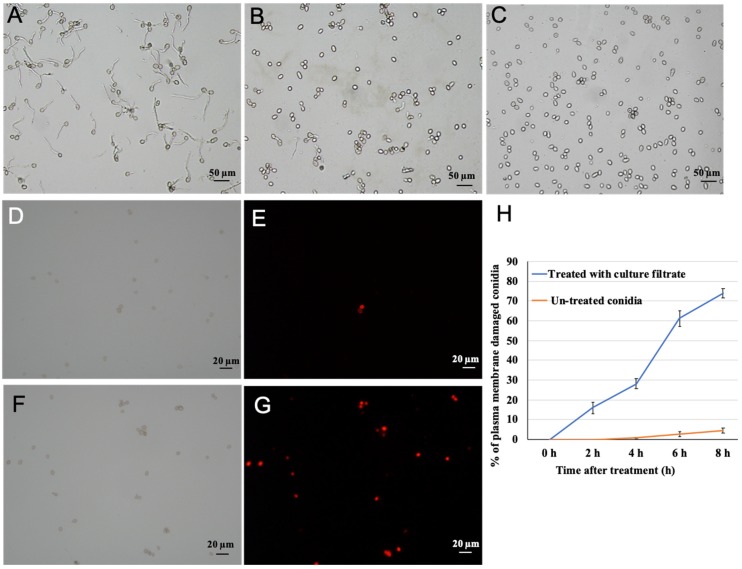
Effect of culture filtrate of *M. anisopliae* on the germination of conidia. (**A**–**C**) *B*. *cinerea* conidia were incubated in sterile water, 1% culture filtrate, and 10% culture filtrate for 8 h, respectively; (**D**,**E**) *B*. *cinerea* conidia was incubated in sterile water for 8 h and then stained by propidium iodide; (**F**,**G**) *B*. *cinerea* conidia was incubated in 10% culture filtrate of *M. anisopliae* for 8 h, and then stained by propidium iodide; (**H**) the percentage of plasma membrane damaged conidia after treatment with culture filtrate of *M. anisopliae*.

Conidia are primary sources of infection for gray mold occurrence. Hence, examining the culture filtrate effect on the germination of conidia can directly verify whether the plant disease can develop and prevail. Therefore, to assess the antifungal efficacy of *M. anisopliae* to *B*. *cinerea* conidia, we evaluated conidia germination, as well as germ tube expansion of *B*. *cinerea* using four concentrations of *M. anisopliae* culture filtrate. All of the tested concentrations of culture filtrate significantly inhibited conidia germination, and the inhibition of conidia germination positively interacted with the culture filtrate concentration. The conidia germination percentage was above 90% in the control at 8 hpi (Figure 4A), while approximately 4.2% of conidia germinated in 1% culture filtrate at 8 hpi, and the germ tube length (average 5.8 µm) of the germinated conidia was much shorter than that of the control *B. cinerea* (average 25.4 µm) (Figure 4B). Notably, no geminated conidia were detected (100% inhibition) in 2% to 10% culture filtrate at 8 hpi (Figure 4C). This suggests that *M. anisopliae* culture filtrate plays a vital role in inhibiting conidia germination, as well as influencing the germ tube length of *B*. *cinerea*.

To examine the possible mechanisms of *M. anisopliae* culture filtrate that show conidia germination inhibition, the condition of the plasma membrane of *B*. *cinerea* conidia was inspected (Figure 4D–H). The plasma membranes damaged conidia discharged red fluorescence beneath the fluorescence microscope, whereas no fluorescence could be distinguished from conidia with undamaged plasma membranes (Figure 4D–G). The 10% culture filtrate of *M. anisopliae* showed 74% damage of the plasma membrane integrity of *B. cinerea* conidia at 8 hpi as compared with a water control. Meanwhile, the membrane veracity of conidia declined rapidly with an increased incubation period (Figure 4H). 

### 2.5. The Antifungal Substance Produced by M. anisopliae Retains Its Activity at High Temperatures 

**Figure 5:**
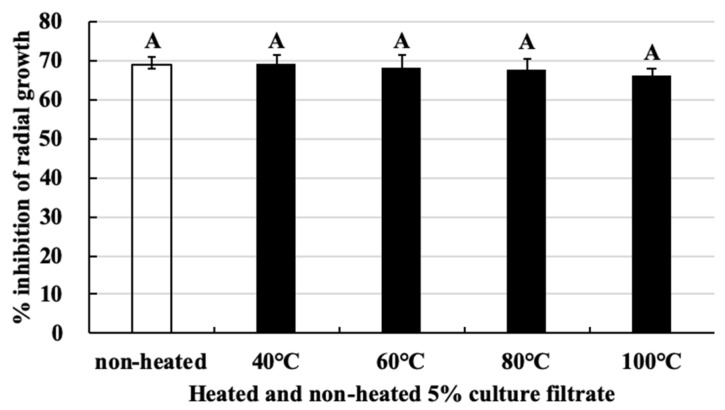
Effect of heating on the antifungal activity of culture filtrate of *M. anisopliae* against *B. cinerea* growth. Bars with the same letters are not statistically significant based on the least significant difference (LSD) test (*P* < 0.01).

The culture filtrate of *M. anisopliae* was heated to 40, 60, 80, and 100 °C. Then, *B. cinerea* was cultured on PDA added heat-treated or non-treated culture filtrate and incubated at 20 °C for 4 days. The heat-treated and non-treated culture filtrates of *M. anisopliae* exhibited parallel inhibitory effects on *B. cinerea* growth, and no significant differences were observed among the treatments (Figure 5). The inhibition effect of 5% non-heated culture filtrate showed 68.65% growth inhibition, as well as 69.1%, 68.31%, 67.4%, and 66.1% growth inhibition when the culture filtrate was heated to 40, 60, 80, and 100 °C, respectively (Figure 5). This shows that the antifungal substance produced by *M. anisopliae* is able to retains its activity at high temperatures.

### 2.6. The Culture Filtrate of M. anisopliae Reduced the Gray Mold Severity of Detached Leaves and Fruits of the Tomato

**Figure 6:**
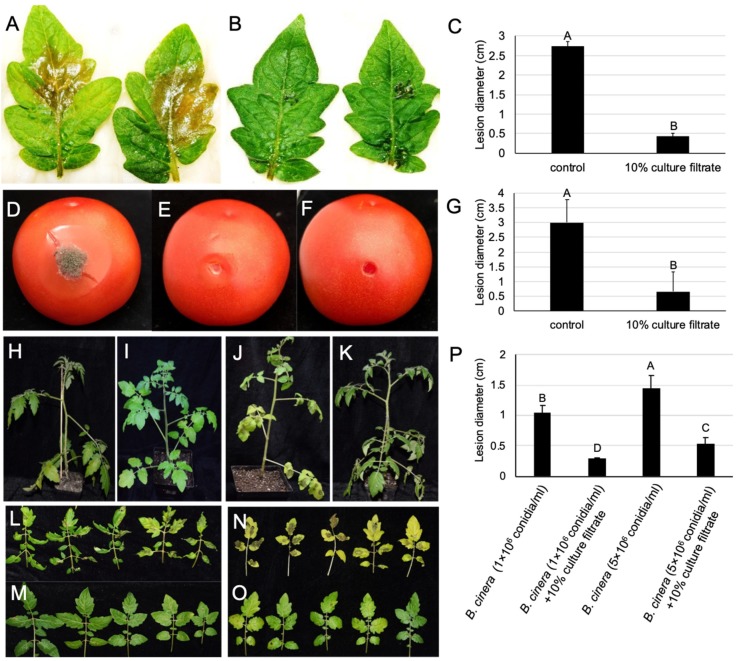
Control of *B. cinerea* on detached tomato leaves, fruits, and whole plants. (**A**–**C**) Effect of *M. anisopliae* culture filtrate on disease severity caused by *B. cinerea*, 4 dpi, 20 °C. Gray mold severity on detached tomato leaves treated with (**A**) sterile water and (**B**) culture filtrate of *M. anisopliae*, (**C**) lesion diameter on detached leaves; (**D**–**G**) efficacy of *M. anisopliae* culture filtrate on tomato fruit mold caused by *B. cinerea*. (**D**) treated with PDB and conidia of *B. cinerea* as a control, (**E**) tomato treated with culture filtrate of *M. anisopliae* and conidia of *B. cinerea,* and (**F**) tomato treated with culture filtrate only; (**G**) lesion diameter on tomato fruits; (**H**–**O**) disease symptoms caused by *B. cinerea* on whole tomato plants, 4 dpi, 20 °C. (**H**,**L**) tomatoes inoculated with 1 × 10^6^ conidia/mL conidia suspension of *B. cinerea*, (**I**,**M**) tomatoes treated with 1 × 10^6^ conidia/mL conidia suspension of *B. cinerea* and culture filtrate of *M. anisopliae*; (**J**,**N**) tomatoes inoculated with 5 × 10^6^ conidia/mL conidia suspension of *B. cinerea*; (**K**,**O**) tomatoes treated with 5 × 10^6^ conidia/mL conidia suspension of *B. cinerea* and culture filtrate of *M. anisopliae*; (**P**) lesion diameter on leaves of whole plants. Bars with the same letters are not statistically significant based on the LSD test (*P* < 0.01).

Detached tomato leaves soaked in sterile water, as well as culture filtrate and inoculated with conidia of *B. cinerea,* exhibited typical lesions (Figure 6A,B). The lesion area of *B. cinerea* on leaves soaked in sterile distilled water was about 2.7 cm at 4 dpi, whereas 10% *M. anisopliae* culture filtrate treatment showed lesions with a diameter of 0.4 cm (Figure 6C). The potential of *M. anisopliae* to control gray mold was also tested on tomato fruits (Figure 6D–F). No lesions were observed when tomato fruits were inoculated with only culture filtrate of *M. anisopliae* (Figure 6F), whereas 4 days after inoculation with *B. cinerea* conidia, the lesion diameter on culture filtrate treated tomato fruits was 0.7 cm, which was only lower than *B. cinerea* conidia treated fruits with a lesion diameter of 3.0 cm (Figure 6G). This suggests that *M. anisopliae* significantly reduced the lesions caused by *B. cinerea* on both leaves and fruits. Furthermore, the biocontrol potentiality of culture filtrate was evaluated on whole plants (Figure 6H–P). The biocontrol efficiency was shown to be 72.38% and 63.19% when plants were treated with a 10% dilution of *M. anisopliae* culture filtrate against 1 × 10^6^ conidia/mL and 5 × 10^6^ conidia/mL of *B. cinerea* as compared with the control. Moreover, when plants were inoculated with only *B. cinerea* spores, plants inoculated with 5 × 10^6^ conidia/mL showed significantly more leaf lesions as compared with those inoculated with 1 × 10^6^ spore/mL (Figure 6L,N).

### 2.7. The Volatile Organic Compounds of M. anisopliae Reduced the Gray Mold Severity of Postharvest Tomatoes

**Figure 7:**
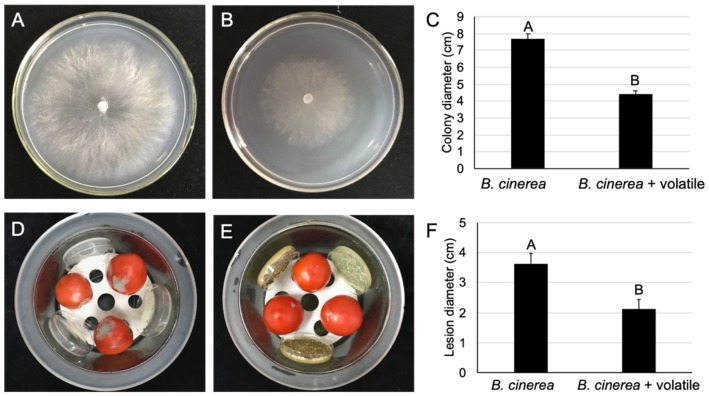
Effect of volatile organic compounds of *M. anisopliae* on *B. cinerea*. (**A**–**C**) Effect of volatile organic compounds of *M. anisopliae* on the growth of *B. cinerea* on PDA, 20 °C, 4 dpi. (**A**) normal colony growth of *B. cinerea*, (**B**) growth inhibition by volatile organic compounds of *M. anisopliae*, and (**C**) colony diameter of *B. cinerea*; (**D**–**F**) the potential of volatile organic compounds of *M. anisopliae* to act on postharvest tomato. (**D**) tomato fruits treated with sterile water, (**E**) tomato fruits treated with volatile organic compounds of *M. anisopliae*, and (**F**) lesion diameter of volatile organic compounds on treated and untreated tomatoes. Bars with the same letters are not statistically significant based on the LSD test (*P* < 0.01).

The impact of volatile organic compounds produced by *M. anisopliae* against the radial growth of *B. cinerea* was checked based on the double-plate chamber method. The results showed that volatile organic compounds produced by *M. anisopliae* inhibited about 42.6% of *B. cinerea* radial growth at 4 days post combined culture (Figure 7A–C). In addition, the volatile organic compounds (VOCs) of *M. anisopliae* reduced the gray mold severity of postharvest tomato by about 41.16% (Figure 7D–F). An average lesion size of 2.1 cm on tomato fruit treated with volatile organic compounds was shown at 4 dpi, while a lesion size of 3.6 cm was recorded in control fruits. Therefore, volatile organic compounds of *M. anisopliae* have the ability to protect postharvest tomatoes against *B. cinerea*.

## 3. Discussion

*B. cinerea* is among the top 10 fungal pathogens. Fungicide application remains the most common method to control *B. cinerea*, and fungicides specifically targeted against *Botrytis* (“botryticides”) represent 10% of the world fungicide market [33]. Fungicide residues lead to risks of environmental pollution and the appearance of multiple fungicide-resistant *B. cinerea* strains [10,11,12]. Some beneficial microorganisms are able to inhibit gray tomato mold, such as *Trichoderma*, *Bacillus*, and *Ulocladium* [34,35], and these could be developed as new biocontrol agents to reduce the use of synthetic fungicides. In the present study, the biocontrol potentiality of *M. anisopliae* strain IBCCM321.93 against gray mold was evaluated. To our knowledge, no previous information is available about the biocontrol efficacy of *M. anisopliae* against gray mold of tomato plants in green houses or mold of postharvest tomato fruits.

In a previous report, *M. anisopliae* strain SD3 produced am inhibition zone in front of *B. cinerea* colony of only about 1 mm [31]. In contrast, *M. anisopliae* strain IBCCM321.93, which was used in this study, was able to produce an inhibition zone of about 6.3 mm in front of *B. cinerea*. It is suggested that *M. anisopliae* strains have diverse biological characteristics and different levels of biocontrol potential. We found that, in addition to inhibiting mycelium growth, the culture filtrate of *M. anisopliae* strain IBCCM321.93 can suppress sclerotia formation and germination, as well as conidia germination. Both sclerotia and conidia play essential roles in disease development in *B. cinerea*. The sclerotia of *B. cinerea* can survive under adverse environmental conditions for a long time without a host, and conidia are a vital source of primary infection for the development of gray mold diseases [33]. Thus, it is possible to control *B. cinerea* by affecting the formation or survival of sclerotia or conidia. Hence, we conclude that *M. anisopliae* may be highly effective for controlling *B. cinerea*. This hypothesis was confirmed by the application of *M. anisopliae* on tomato plants and fruits. We found the cultural filtrate of *M. anisopliae* performs biocontrol efficacy against gray mold on detached tomato leaves, whole plants, as well as postharvested tomato fruits. However, the preventive control effect of culture filtrate of *M. anisopliae* against gray mold of tomato is still unknow. It could be evaluated in future by treating plants with culture filtrate before plants are infected naturally by *B. cinerea*. It has been reported that *M. anisopliae* is capable of endophytic colonization on the roots of plants, thereby enhancing plant growth and acting as a plant disease antagonist [36]. The *M. anisopliae* agent used in this study was culture filtrate, suggesting that the mechanism of *M. anisopliae* that controls *B. cinerea* possibly depends on secondary metabolites other than endophytic colonization.

The influence of the cultural filtrate of *M. anisopliae* on *B. cinerea* was observed in this study. Transmission electron microscopy showed that the cell organelles of *B. cinerea* mycelia treated with *M. anisopliae* culture filtrate were seriously damaged, suggesting that *M. anisopliae* produces some active substance with toxicity action against *B. cinerea*. In addition, based on fluorescence emissions from conidia treated with propidium iodide, it is assumed that some secondary metabolites generated by *M. anisopliae* also play a role in damaging the plasma membrane integrity of conidia. Therefore, we assume that the damage of cell organelles and plasma membrane could be the main method by which *M. anisopliae* suppresses the normal development of *B. cinerea*. In a previous report, several mechanisms were investigated to describe the mode of action accountable for the insecticide activity of *M. anisopliae*. It was stated that the secretion of protease, chitinase, and lipases by *M. anisopliae* are associated with the process of infection [37]. These enzymes are considered to assist host tissue penetration by degrading the host’s exterior layers (cuticles of nematode, insects, and cell wall of fungi) or utilizing the host’s proteins for nutrition [38]. For example, previous studies reported that chitinase from *Trichoderma harzianum* and *Clonostachys rosea* enhanced the inhibition of *B. cinerea* [39,40,41,42]. In this study, the antifungal substance produced by *M. anisopliae* retained its activity after high-temperature treatment, suggesting that the main part of the antifungal substances are probably not proteins. This property of the antifungal substance indicates that the biocontrol agent produced by *M. anisopliae* can be stocked at room temperature. In addition, it can ensure the stability of biocontrol agents under high temperatures, such as in summer. Therefore, this feature can reduce the storage cost and expand the application area of the biocontrol agent. It has been reported that *M. anisopliae* also produces secondary metabolites, such as destruxins, swainsonine, and polyketides [43]. Whether these or some other new substances play roles in the inhibition of *B. cinerea* still requires study. Cultural filtrate has antimicrobial activity. To our knowledge, this work experiment reports, for the first time, that volatile compounds produced by *M. anisopliae* possess antifungal activity. The volatile organic compounds produced by *M. anisopliae* also act as repellents against insects such as *Macrotermes michaelseni* [44], suggesting the *M. anisopliae* has the potential to be developed as a fumigant to control both plant diseases and insects. However, whether the substances play roles in antifungal activity in the volatile organic compounds requires further study.

## 4. Materials and Methods

### 4.1. Fungal Isolates and Cultural Condition

*M. anisopliae* strain IBCCM321.93 used in this study was the same as that used in the previous report [45]. *M. anisopliae* was cultured on potato dextrose agar (PDA) medium at 28 °C for 2 weeks. 

*B. cinerea* strain B05.10 was incubated at 20 °C on PDA in the dark for 3 to 4 days to determine radial growth rates and for 20 days to determine sclerotia production [46]. The conidia of this strain were produced following a modified method that was described in the previous report [47,48]. Fifteen days post-incubation (dpi) on PDA medium, the conidia were harvested by flooding the plate with sterile water (plus 0.5% Tween 80, Xilong Scientific Co., Ltd., Guangdong, China), and the required conidia concentration was adjusted with hematocytometer. 

### 4.2. Assay Inhibitory Effect of M. anisopliae

The inhibitory effect of *M. anisopliae* against *B. cinerea* was detected using a dual culture technique on the PDA plate. One mycelial plug (6 mm diameter) of *M. anisopliae* was placed on a new PDA plate 1 cm away from the border of every petri dish, and this was incubated at 28 °C for two days. Then, a mycelial plug (6 mm diameter) of *B. cinerea* derived from the edge of the vigorously growing colony was put on the other side of the plate. All plates with *M. anisopliae* and *B. cinerea* were incubated at 20 °C for 4 days until a clear inhibition zone was quantified. The trial was repeated three times with three replications. Inhibition of the pathogen development was assessed by means of the formula IG (%) = (BCK − BCF)/BCK × 100%, where IG denotes the growth of inhibition, BCK indicates the colony width in the control, and BCF indicates the colony width after treatment with culture filtrate, respectively [49].

### 4.3. Preparation of the Culture Filtrate of M. anisopliae

One block of a 14-day-old *M. anisopliae* mycelia plug (5 mm diameter) was inoculated in 250 mL conical flasks containing 100 mL of potato dextrose broth (PDB) (pH 6 to 6.5) and cultured in a shaker for 10 days at 160 rpm and 28 °C. Then, the fermented broth was centrifuged at room temperature at 12,000 rpm for 15 min to remove the mycelium. Then, the supernatant fraction was passed through sterile gauze cloth. The culture filtrate was collected after the supernatant passed through a 0.22 μm membrane filter (Millipore Sigma, USA). 

### 4.4. Effect of M. anisopliae Culture Filtrate against the Growth of B. cinerea 

*B. cinerea* was cultured on PDA and maintained at 20 °C in the dark. Petri dishes with PDA and culture filtrate of *M. anisopliae* were used to assess the effects of culture filtrate upon the radial growth of *B. cinerea*. In the treatment, the final concentrations of the culture filtrate (10%, 5%, 2%, and 1% (*v*/*v*)) were added PDA, whereas sterile PDB mixed with PDA served as a control. A three-day-old mycelial plug (5 mm in diameter) of *B. cinerea* was put in the middle of each petri dish and incubated at 20 °C. Each treatment set had three replications. Four days after incubation, the colony diameter of every dish was recorded in two vertical directions, and the growth inhibition (IG) of *B. cinerea* by the culture filtrate of *M. anisopliae* was calculated using the formula IG = [(mean of colony diameter in control – mean of colony diameter in treatment)/mean of colony diameter in control] × 100 [50]. The experiment was conducted three times.

### 4.5. Effect of Culture Filtrate of M. anisopliae against the Germination of Conidia and Sclerotia of B. cinerea

The inhibitory impacts of the culture filtrate of *M. anisopliae* on the germination of conidia, as well as the germ tube development of *B. cinerea* were tested using the procedures described in a previous report [51]. Germination of conidia was confirmed if the germ tube length was equivalent to or larger than the conidia [52]. The final concentration of the culture filtrate (1%, 2%, 5%, and 10%) was attained with sterile PDB. Fifteen microliters of the distinct dilutions were mixed with 3 µL of *B. cinerea* conidia suspension dosed at 5 × 10^5^ conidia/mL and placed upon a glass slide. Sterilized PDB served as a control instead of culture filtrate. All slides were put into Petri dishes containing moist blotter paper to retain an elevated relative humidity. Then, they were incubated under white radiant light in an evolution chamber at 20 °C for 8 h. Germinated conidia were counted by microscope observation (Olympus CX41, Japan), and the germ tube length was measured. Afterwards, the germination inhibition percentage of conidia was calculated following the method of Youssef et al. [53]. For all treatments, at least 300 conidia were observed to determine the germination percentage, and 50 conidia were used for germ tube dimension when more than 90% of *B. cinerea* conidia in the PDB control were germinated after 8 h of incubation. This experiment was performed twice with three replications. *B. cinerea* was cultured on PDA at 20 °C for 21 d. To test the influence of *M. anisopliae* on the sclerotia germination of *B. cinerea*, black matured sclerotia were used in the assay following the method described by Yang et al. [54]. The surface-sterilized sclerotia were dipped in a 10% (*v*/*v*) culture filtrate of *M. anisopliae* for 24 h, before being air-dried and placed into PDA plates. Every treatment was conducted three times with 50 deliberate sclerotia in each treatment. All PDA plates containing sclerotia were incubated at 20 °C for 2 days. Germinated sclerotia were counted in the control and treated groups, and then sclerotia germination rates were calculated.

### 4.6. Effect of Culture Filtrate of M. anisopliae on the Plasma Membrane Integrity of B. cinerea Conidia

The conidia concentration of *B. cinerea* (1 × 10^6^ conidia/mL) was incubated in a 10% culture filtrate of *M. anisopliae* at 20 °C without shaking. Sterile distilled water was used instead of culture filtrate in the negative control. After 2, 4, 6, and 8 h of incubation, conidia specimens were centrifuged at 10,000 rpm. Subsequently, staining and inspections of conidia were performed, as described previously [4]. Concisely, the conidia were dyed in 10 μg/mL of propidium iodide (Sigma-Aldrich, USA) for five min at room temperature and then centrifuged. The conidia pellets were rinsed twice with PBS buffer (pH 7.0). Inspections were conducted with a Nikon Eclipse 80i microscope (Japan) fitted with epifluorescence optics using blue excitation (450 to 490 nm) and 526 nm barrier filters. A total of 300 conidia was checked arbitrarily to determine whether conidia were stained by the dye. Membrane veracity damage was calculated following the formula (MV = (number of total conidia – number of stained conidia)/(number of total conidia) × 100%). This experiment was performed three times.

### 4.7. Heat Stability of Culture Filtrate Activity of M. anisopliae

To investigate the heat stability of culture filtrate, it was exposed to different temperatures (40, 60, 80, and 100 °C) for 15 min. After exposure to heat, culture filtrate of *M. anisopliae* was promptly cooled to room temperature, and its antagonistic activity was assessed against *B. cinerea*. The inhibitory effects of the heat-treated 5% culture filtrates on the radial growth of *B. cinerea* was determined, as described in Section 4.4. This experiment was repeated twice.

### 4.8. Sample Preparation for Transmission Electron Microscopy

The effect of *M. anisopliae* culture filtrate on the cell components of *B. cinerea* mycelia was examined with transmission electron microscopy (TEM). The mycelial morphology of *B. cinerea* was examined following the method described by Zhang with appropriate modifications [55]. Briefly, the sterilized cellophane sheets were set on PDA with 5% culture filtrate of *M. anisopliae*, and PDA plates without culture filtrate were set as the control. Then, three-day-old culture mycelial plugs of *B. cinerea* were inoculated on each plate and cultured at 20 °C for 4 days. Afterwards, the colonized cellophane sheets were sliced into 2 × 3 mm units with a sharp blade. The small units of cellophane sheet were fixed in Karnovsky’s fixative (2% paraformaldehyde and 2.5% glutaraldehyde, (Polysciences, Inc., Warrington, PA) in 0.1 M MOPS buffer, pH 7.0) at 4 °C for 24 h, Then, they were washed in MOPS buffer (pH 7.0, MOPS stands 3-[N-morpholino]propane-sulphonic acid, Sigma- Aldrich, USA) three to four times at 25 °C for 10 min, fixed in 1% osmium tetroxide (Anilax Chemicals Inc. USA) for 2 h, and dyed in 5% (*w*/*v*) uranyl acetate (Thomas Scientific, USA) in 50% (*v*/*v*) ethanol for 1 h. Subsequently, the affixed mycelial samples were desiccated in 30% ethanol for 15 min, 50% ethanol for 15 min, 70% ethanol for 15 min, 80% ethanol for 20 min, 90% ethanol for 20 min, and 100% ethanol for 30 min. Then, 100% acetone was used to dehydrate samples twice, for 30 min each time. Next, samples were embedded with SPI-Pon 812 embedding kit (SPI Supplies, USA), along with polymerization at 60 °C for 12 h. Thin slices (50 to 60 nm) were cut with an ultra-microtome, mounted on copper grids, stained with 2% uranyl acetate and 5% aqueous lead citrate, and examined with a FEI Tecnai G2 transmission electron microscope at 75 kV acceleration voltage. Images were recorded with a 4 KCCD camera (Model, 832 ORIUS, Gatan, Pleasanton, CA, USA).

### 4.9. Effect of Culture Filtrate on Gray Mold on Detached Tomato Leaves

Healthy tomato leaves were collected from five-week-old plants (*Lycopersicon esculentum* cv. Mill) and surfaces were disinfected by soaking in a 1% NaClO solution for 2 min (Sino-pharm Chemical Reagent Co., Ltd., Shanghai, China). Then, leaves were thoroughly washed with sterilized, distilled water and dried naturally. Whole leaves were soaked in 10% culture filtrate for 30 min and dried inside a laminar airflow. Subsequently, sterile water soak, instead of culture filtrate, was used as control. After drying the detached leaves’ surfaces, 15 μL of *B*. *cinerea* conidia suspension (1 × 10^6^ conidia/ml) in 0.5% Tween 80 comprising 1 mg/mL of sucrose was dripped for inoculation. Each detached leaf was incubated inside the incubator at 20 °C along with the white light. Four days after incubation, lesion diameters were measured, and the biocontrol efficacy of *M. anisopliae* was calculated as (lesion diameter in the control − lesion diameter in the treatment)/lesion diameter in the control × 100 [56]. This experiment was performed twice.

### 4.10. Effect of Culture Filtrate of M. anisopliae on the Gray Mold of Ripe Tomato Fruits

In this assay, ripe tomato fruits were infected as described in [57]. Mature, red, rot-free tomato fruits, even in size and injury, were used in the gray mold biocontrol assays. The surfaces of the ripe tomatoes were sterilized with 1% (*w*/*v*) NaClO solution for 2 min, and then washed with water and naturally dried. Disinfected tomato fruits were wounded (2 mm depth and 2 mm in diameter) by forceps. Then, 30 μL of 10% culture filtrate of *M. anisopliae*, or sterile PDB instead of culture filtrate as the control, was placed into the wounds. Approximately two hours after treatment, 15 μL of *B*. *cinerea* conidia (1 × 10^6^ conidia/ml) suspension was inserted into each wound. All tomato fruits were put on moist absorbent paper in plastic cases to retain a high level of humidity, and these were incubated at 20 °C. The lesion diameters of the infected tomato fruits were measured after 4 days of incubation in accordance with Liu et al. [58]. Every treatment comprised 10 tomatoes, and the trial was conducted twice. 

### 4.11. Effect of Culture Filtrate of M. anisopliae against Tomato Plant Gray Mold

Tomato seeds (*Lycopersicon esculentum* cv. Mill) were sown in vermiculite soil and transplanted after two weeks in an individual pot containing potting mix soil. Plants were cultured in the greenhouse for 6 weeks. Afterwards, each leaf of five leaflets was sprayed with 10% culture filtrate until overflow 4 h prior to inoculation with *B. cinerea* conidia. Leaflets sprayed with 10% PDB medium served as a control. Subsequently, 25 leaves per plant were inoculated with 15 µL of conidial suspension (in 1% sucrose solution containing 0.1% Tween 80), and 0.1% Tween 80 containing sterile distilled water was used as a control. Two *B. cinerea* conidia concentrations (1 × 10^6^ and 5 × 10^6^ conidia/mL) were used for inoculation in the assays. The experimental plants were organized in a complete randomized block design with two replicates of three plants for each treatment. After 4 days of inoculation with *B. cinerea* conidia, the gray mold incidence was recorded. The biocontrol efficacy was estimated using the gray mold infection of detached leaves, as previously mentioned. 

### 4.12. Volatile Effect of M. anisopliae on B. cinerea

A *M. anisopliae* mycelium plug (6 mm diameter) was placed on a PPDA (potato peptone dextrose agar; 200 g potato juice, 10 g peptone, 20 g dextrose, and 15 g agar in 1 liter) plate and cultured at 28 °C. Seven days later, the petri dish containing *M. anisopliae* was covered by another *B. cinerea* inoculated PDA plate, and two plates were sealed with parafilm to get a double-plate chamber [19]. A *B. cinerea* inoculated PDA plate covered with a fresh PPDA plate served as the control. Double plates were incubated in a growth chamber at 20 °C. Four days after incubation, the *B. cinerea* colony diameters were measured in treated and control treatments, and the inhibition percentage of radial growth was assessed. For each treatment, three replicates and three repeats were performed. 

### 4.13. Effect of M. anisopliae VOCs on Botrytis Gray Mold of Postharvest Tomatoes 

The assay was conducted in sealed glass desiccators following the method described by Huang et al. [59]. *M. anisopliae* was cultured on PPDA at 28 °C for 7 days. The lids of plates with *M. anisopliae* were removed, and then one plate (16 cm) was positioned at the bottom, and three plates (9 cm) were vertically positioned on the pierced ceramic board of each desiccator. For the control treatment, uncovered PPDA plates without strains were placed in the desiccator. Surface sterilized healthy tomatoes were wounded (2 × 2 mm, depth and diameter) with sterile forceps, inoculated with 15 μL of *B. cinerea* conidial suspension (1 × 10^6^ conidia/mL), and put on the pierced ceramic clapboard in the desiccator. All inoculated tomatoes containing desiccators were independently covered with a lid and kept at 20 °C. The lesion diameter of the infected tomatoes was measured 4 days after incubation, in accordance with Liu et al [58]. Nine tomato fruits were used in each treatment, and the trial was conducted twice.

### 4.14. Data Analysis

Data were statistically analyzed with a one-way analysis of variance (ANOVA) using SAS software (SAS Institute, Cary, NC, USA, v. 8.1, 1999). The data are expressed as means ± standard error (SE). Treatment means were partitioned using the least significant difference (LSD) test at *P* < 0.01.

## 5. Conclusions

In this research, it was found that, in addition to pest control ability, the entomopathogenic fungus *M. anisopliae* can inhibit *B. cinerea* mycelia growth, sclerotia formation and germination, and conidia germination. Additionally, both the culture filtrate and volatile compounds produced by *M. anisopliae* possess antifungal activity, and the culture filtrate retains its activity under high temperatures. The results of the present study indicate that *M. anisopliae* is a potential alternative to chemical fungicides for controlling *B. cinerea* on pre- and postharvest crops. However, a deeper understanding of the antifungal mechanism in relation to the structure–activity relationship of the active substance could be developed in more depth in the future. In addition, the potential of *M. anisopliae* biocontrol on other important plant pathogens could be tested in the future.

## Figures and Tables

**Table 1:** Effect of culture filtrate of *M. anisopliae* on *B. cinerea* sclerotia germination.

Treatment	Average Germination Rate (%)	% Relative Inhibition of Sclerotia Germination
Control	96.89 ± 2.13 A	-
10% culture filtrate treated	35.04 ± 1.69 B	63.85 ± 1.16

Note: Data represents mean ± SE. Same letters in different columns are not significantly different according to LSD *t*-tests at the *P* < 0.01 level.
